# Release from Cross-Orientation Suppression Facilitates 3D Shape Perception

**DOI:** 10.1371/journal.pone.0008333

**Published:** 2009-12-16

**Authors:** Andrea Li, Qasim Zaidi

**Affiliations:** 1 Department of Psychology, Queens College, City University of New York, Flushing, New York, United States of America; 2 Graduate Program in Vision Science, State University of New York College of Optometry, New York, New York, United States of America; University of California Davis, United States of America

## Abstract

Cross-orientation suppression (COS) in striate cortex has been implicated in the efficient encoding of visual stimuli. We show that release from COS facilitates the decoding of 3-D shape. In planar surfaces overlaid with textures, slanting the surface can increase the visibility of the component parallel to the slant. Since this component provides the orientation flows that signify 3-D shape, the enhancement of visibility facilitates 3-D slant perception. Contrast thresholds reveal that this enhancement results from a decrease in COS when 3-D slant creates a frequency mismatch between texture components. We show that coupling compressive nonlinearities in LGN neurons with expansive nonlinearities in cortical neurons can model the frequency-specific component of suppression.

## Introduction

**Figure 1:**
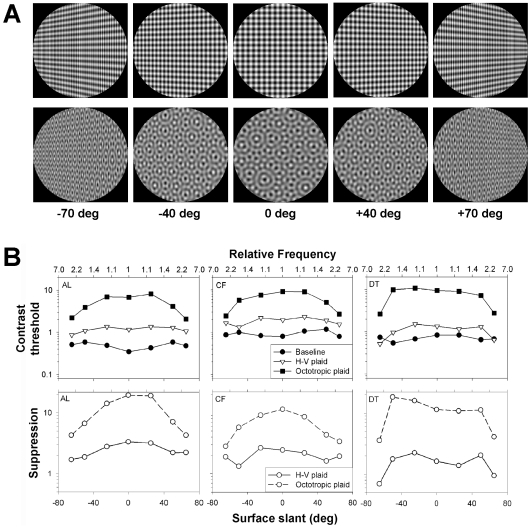
Suppression of the test grating as a function of surface slant. A. Planar surfaces at different slants patterned with horizontal-vertical (h–v) (top) and octotropic (bottom) plaids. B. Top: Contrast thresholds for three observers for the horizontal component alone (filled circles), with a vertical component (open triangles), and with seven non-horizontal components (filled squares) as a function of surface slant. The top axis of each panel represents the frequency of the vertical component relative to the frequency of the test in the image. Bottom: Suppression factor as a function of surface slant.

In the perspective image of a slanted textured surface, oriented components of the texture that are aligned with the 3-D slant converge to form orientation flows ([1], [2], [3]), while components orthogonal to the slant increase in frequency (Figure 1a). On casual observation, the horizontal component appears perceptually more salient than other components when a surface is slanted (Figure 1a, top left and right) than it does when the surface is parallel to the frontal plane (Figure 1a, top center). The increase in saliency is more pronounced in complex texture patterns, e.g. the octotropic plaid, which consists of eight gratings of the same frequency, equally spaced in orientation (Figure 1a bottom). Since these converging orientation flows play a critical role in conveying the perceived 3-D slant and shape of the surface ([3], [4], [5], [6]), an increase in their saliency should enhance the 3-D perceived slant. The goal of this work is to examine the neural mechanisms that enhance the visibility of orientation flows.

Many surface textures contain components of roughly the same frequencies at many different orientations, with most of the frequencies in the higher frequency declining segment of the human CSF ([7]). Slanting the surface increases the frequencies of components not aligned with the slant ([8]), thus leading to a reduction in visibility. If different oriented components were processed independently by the visual system, the increase in saliency of the components parallel to the slant could be due just to the reduced visibility of the other components. However independent processing of different orientations is not a feasible premise.

The response of oriented neurons in cat and primate striate cortex to a stimulus at a preferred orientation is suppressed by the superposition of a second oriented stimulus, even at the null orientation. Parallel to these results, psychophysical studies have reported that the contrast threshold of an oriented stimulus is increased in the presence of a superimposed orthogonal stimulus. Physiologically measured cross-orientation suppression (COS) is broadband for orientation and occurs over a wide range of spatial frequencies ([9], [10], [11]). Psychophysically measured COS appears to be broadband for orientation ([12]), but with mixed evidence for frequency-selectivity ([13], [14], [15], [16], [17]). Thus it is possible that psychophysically measured COS has components that are distinct from the COS measured in V1 neurons.

In this study we identify the mechanism underlying the change in salience of orientation flows. In the first experiment, we show that the visibility of orientation flows increases as a function of surface slant. In the second experiment, we show that the increased salience results from the frequency-selectivity of COS and not the frequency dependent visibility of the masking components.

## Methods

All research followed the tenets of the World Medical Association Declaration of Helsinki and informed consent was obtained from the subjects after explanation of the nature and possible consequences of the study. The research was approved by the Queens College Institutional Review Board.

### 1. Apparatus and Presentation

Stimuli were presented on a 22″ Mitsubishi Diamond Pro 2070 flat screen CRT monitor with an 1024×768 pixel screen running at a refresh rate of 100 Hz via a Cambridge Research Systems ViSaGe Visual Stimulus Generator controlled through a 3.2 GHz Pentium 4 PC. Observers' head positions were fixed with a chinrest situated 1 m from the stimulus monitor. All stimuli were presented so that the center of each image was level with the observer's eye. Viewing was monocular in a dimly lit room, and there was no feedback.

### 2. Stimuli and Procedure

Planar surfaces were patterned with horizontal-vertical (h–v) and octotropic plaid patterns and projected in perspective. All stimuli were presented such that the horizontal grating component was interleaved with non-horizontal components in alternating frames at 100 Hz. This technique enabled us to alter the contrast of the horizontal component independently from the other components. For the h–v plaid, the contrast of the vertical grating was fixed at 50%, the highest possible for interleaved frames. Similarly, the contrast of each of the non-horizontal gratings in the octotropic plaid was fixed at the highest possible level, 7.1%. The phases of the plaid pattern components were randomized on each trial. Stimuli were presented in circular apertures spanning 6.5 deg against a grey background at the mean luminance of 58 cd/m^2^.

Contrast thresholds of the horizontal component were determined using a 2IFC paradigm. Each session was preceded by a grey screen with a central black fixation cross that remained onscreen for 1 minute. The fixation cross remained onscreen for the duration of the session. After the initial adaptation, a tone signaled the start of the trials. Each of the two stimulus intervals in each trial lasted 500 msec, separated by a 400 msec inter-stimulus interval. Audible beeps of different frequencies signaled the presentation of each of the two stimulus intervals. Test contrast was varied in interleaved 3-down/1-up double-random staircases to ascertain the 79% correct point ([18]). Each staircase completed two reversals at 1.8% contrast steps, then eight reversals at 0.4% contrast steps. Threshold was estimated as the average of the last six reversals.

### 3. Experiment 1: Orientation Flow Visibility as a Function of Surface Slant

Surfaces were patterned with 3 cpd h-v and octotropic plaids (Figure 1a). For each of the two plaid types, observers completed eight sessions which were grouped as follows. One baseline session measured contrast thresholds for the horizontal grating alone in the fronto-parallel orientation. Each of three other baseline sessions measured contrast thresholds of the horizontal grating alone at left and right slants of 25, 50, and 65 deg. In the other four sessions, contrast thresholds were measured in the presence of the non-horizontal components. Thus there were a total of 16 sessions per observer. For each pattern type, the four baseline sessions were run first in random order, then the remaining sessions were run in random order. Each session took approximately 10–15 minutes.

### 4. Experiment 2: Frequency-Selectivity of Cross-Orientation Suppression Mechanism

**Figure 2:**
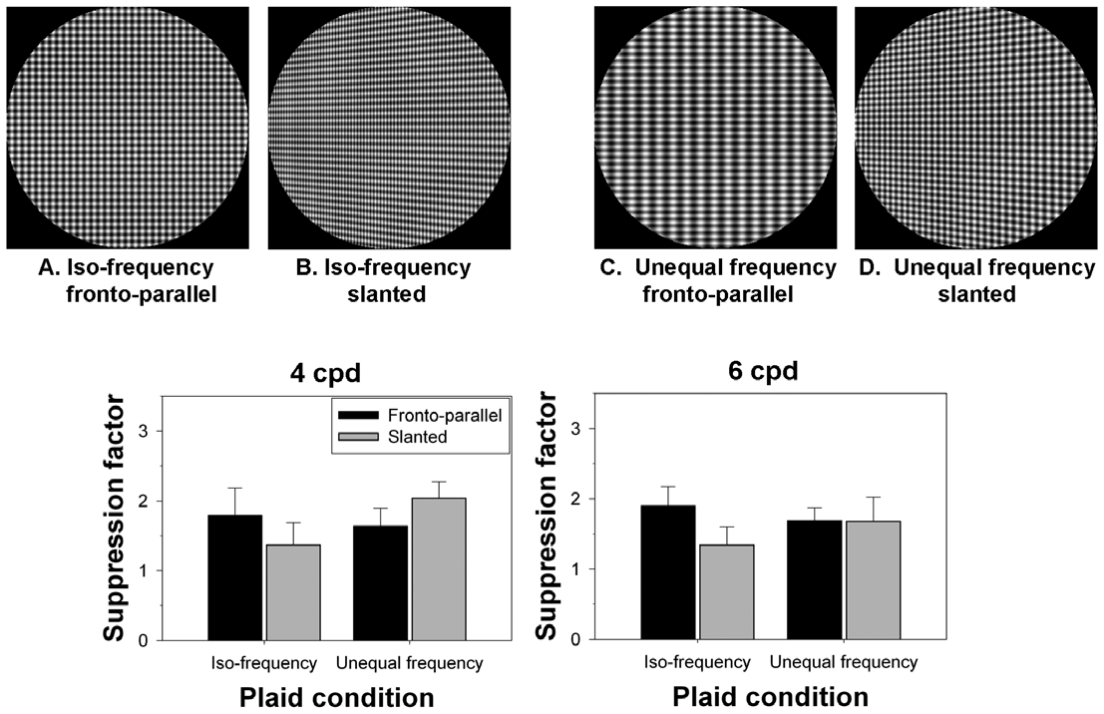
Suppression of the test grating from iso-frequency vs. unequal frequency masks. Top: An iso-frequency plaid (a), and an unequal frequency plaid (c) consisting of a horizontal grating and a vertical grating at half the frequency. At slants of 60 deg, the components in the image of the iso-frequency plaid are unequal in frequency (b), and the components in the image of the unequal frequency plaid are equal in frequency (d). Bottom: Suppression factors averaged across three observers (left: 4 cpd test frequency, right: 6 cpd test frequency). Error bars represent one standard error of the mean. Black bars represent suppression factors in the fronto-parallel conditions, and grey bars represent suppression factors in the slanted conditions.

Fronto-parallel surfaces were patterned with an iso-frequency h–v plaid (Figure 2a) or an h–v plaid consisting of a vertical grating of half the frequency of the horizontal grating (Figure 2c). The same surfaces were also presented slanted at left or right at 60 deg which acts to approximately double the vertical frequency in the image. Consequently, the frequencies in the image of the slanted 6 cpd iso-frequency plaid become 6 cpd horizontal and 12 cpd vertical (Figure 2b) and the frequencies in the image of the 6 cpd horizontal and 3 cpd vertical plaid become approximately equal at 6 cpd (Figure 2d). To test whether suppression is a function of the similarity of frequencies between the test and mask, or of the salience of the mask, we needed to select frequencies from the small set that are highly visible, effectively convey surface slant, and are significantly less salient when doubled. We used frequencies of 4 and 6 cpd which satisfy these requirements by being just past the peak of the human CSF.

Contrast thresholds for the horizontal grating were measured using the interleaved staircase procedures. Observers ran four different sessions, three times each: one baseline session for the horizontal grating alone at fronto-parallel, rightward slanted by 60 deg and leftward slanted by 60 deg orientations, one session for fronto-parallel plaids and two sessions for slanted plaids. The slanted sessions were blocked in order to contain both types of plaids and both types of slants within each session, while keeping the length of sessions similar to the sessions testing the fronto-parallel stimuli.

### 5. Observers

One of the authors and two experienced but uninformed observers participated in this study. All had normal or corrected-to-normal visual acuity.

## Results

### 1. Experiment 1: Orientation Flow Visibility as a Function of Surface Slant

Contrast thresholds of the horizontal components in the different conditions are shown for the three observers in Figure 1b in separate columns. The panels in the top row plot contrast thresholds of the horizontal grating alone (filled circles), in the presence of the vertical grating in the h–v plaid (open triangles), and in the presence of the seven non-horizontal components in the octotropic plaid (filled squares) as a function of surface slant. The axis along the top of each panel represents the frequency of the vertical grating component as it changes with surface slant relative to the frequency of the test. Thresholds of the grating alone (filled circles) are relatively unchanged by surface slant, reflecting the fact that the spatial frequency of this component is relatively unchanged. The presence of the vertical grating (open triangles) increases thresholds for all surface slants (except for the steepest slants for observer DT), reflecting an overall decrease in visibility of the horizontal component. Thresholds increased even more in the presence of the seven non-horizontal components of the octotropic plaid (filled squares).

We quantified the suppression induced by non-horizontal components by dividing thresholds of the horizontal grating in the presence of other components by thresholds in the absence of other components ([16]). The suppression factors for the simple and octotropic plaids are plotted as functions of surface slant, with solid and dashed lines respectively, in the bottom panels of Figure 1b. Suppression for both patterns decreases as surface slant increases, with substantially greater and steeper changes in suppression for the octotropic plaid.

We have previously shown that perceived orientation flows determine the perception of 3-D shape from texture ([3], [5], [19], [20]). 3-D shape is not perceived when the flows are physically present if they are masked by other components (see [5], Figure 10). The results in Figure 1 indicate that orientation flows are more visible for the h–v than the octotropic plaid at shallow slants, but equally visible at steep slants. Hence, slants should be easier to see for the h–v plaid than the octotropic plaid at shallow angles, but the two should be equally perceptible at steep angles. This prediction is borne out in Figure 1 where the orientation flows and thus slants are easier to see in the h–v plaid than the octotropic plaid at ±40 deg, but are equally visible for the two plaids at ±70 deg.

### 2. Experiment 2: Frequency-Selectivity of Cross-Orientation Suppression

It is clear from the results in Figure 1 that contrast thresholds are raised by orthogonal masks, which is a signature of COS. Since the frequencies in the fronto-parallel plane were 3 cpd which is near the peak of the human CSF, the question remains whether the peak suppression is a function of the similarity of frequencies between the test and mask, or of the salience of the mask.

The four conditions of Experiment 2 provided two independent comparisons of these hypotheses. In Figure 2 (bottom), mean suppression factors averaged across the three observers are plotted for all conditions for the 4 cpd horizontal grating (left) and the 6 cpd horizontal grating (right). Error bars represent one standard error of the mean. Data in each panel are plotted in the same order from left to right as the four stimulus conditions shown above. First, the similarity hypothesis predicts that thresholds should be higher in the iso-frequency fronto-parallel plaid (Figure 2a) than for the unequal frequency fronto-parallel plaid (Figure 2c), whereas the salience hypothesis predicts the opposite. Thresholds for both the 4 cpd and 6 cpd test gratings were raised more by the iso-frequency mask than the more salient unequal frequency mask. Second, the increase in suppression for the 4 cpd condition when the unequal frequency plaid (Figure 2c) is slanted (leading to an iso-frequency image pattern, Figure 2d) also supports the similarity hypothesis over the salience hypothesis. In addition, in comparing the two slanted plaids, suppression was greater when the image pattern was iso-frequency (Figure 2d) than when the surface pattern was iso-frequency (Figure 2b). Since we expected suppression to decrease with increasing slant for the iso-frequency condition and increase with slant for the unequal frequency condition, we tested for interaction between the frequency conditions and the slant conditions in a 2×2 ANOVA. The interaction was in the correct direction for both spatial frequencies, and statistically significant at the .05 level for the 4 cpd test (F(1,12) = 23.12, p = .0406) but not for the 6 cpd test (F(1,12) = 12.00, p = .0742).

These results indicate that the COS from the vertical grating is greatest when the frequency in the projected image is equal to that of the horizontal grating, even when the frequency is one to which we are less sensitive. Previous measurements of the spatial frequency tuning of COS ([21]) showed a decrease in masking for a 4 cpd test when the mask frequency increased from 4 to 8 cpd, but did not determine whether spatial-frequency mismatch or a decrease in mask saliency was the cause.

### 3. Feed-Forward Models of Cross-Orientation Suppression

COS is well-documented in cortical area V1, the first site in the visual pathway containing orientation tuned cells. COS has been attributed to compressive contrast nonlinearities in LGN ([22], [23]), but a cortical component has also been revealed ([24]). Although several electrophysiological studies examining the frequency selectivity of COS suggest that suppression mechanisms are broadly tuned ([11], [25], [26]), it is unclear whether this kind of tuning plays out psychophysically. It would be remarkable if the facilitation of 3-D shape perception occurs automatically through the neural processes that lead to COS, so to ascertain its locus, we have explored the possibility of frequency selectivity in an LGN based model.

Although intra-cortical inhibition was the original suggestion for COS, the fact that suppression is not reduced by prior monocular or binocular adaptation to the masking stimulus, that suppression is robust for masks at temporal frequencies beyond the limits of cortical neurons, and that COS has an early onset led to the suggestion that the suppression results from the depression of thalamo-cortical synapses ([27], [15], [28]). More recent papers quantifying the fast recovery times of COS ([22]) and the suppression of both synaptic inhibition and excitation by orthogonal masks ([23]) challenge the notion of synaptic depression. Instead these models suggest that COS results from contrast saturation and rectifying nonlinearities in the LGN, and expansive spike threshold nonlinearities in the cortex ([22], [23]).

**Figure 3:**
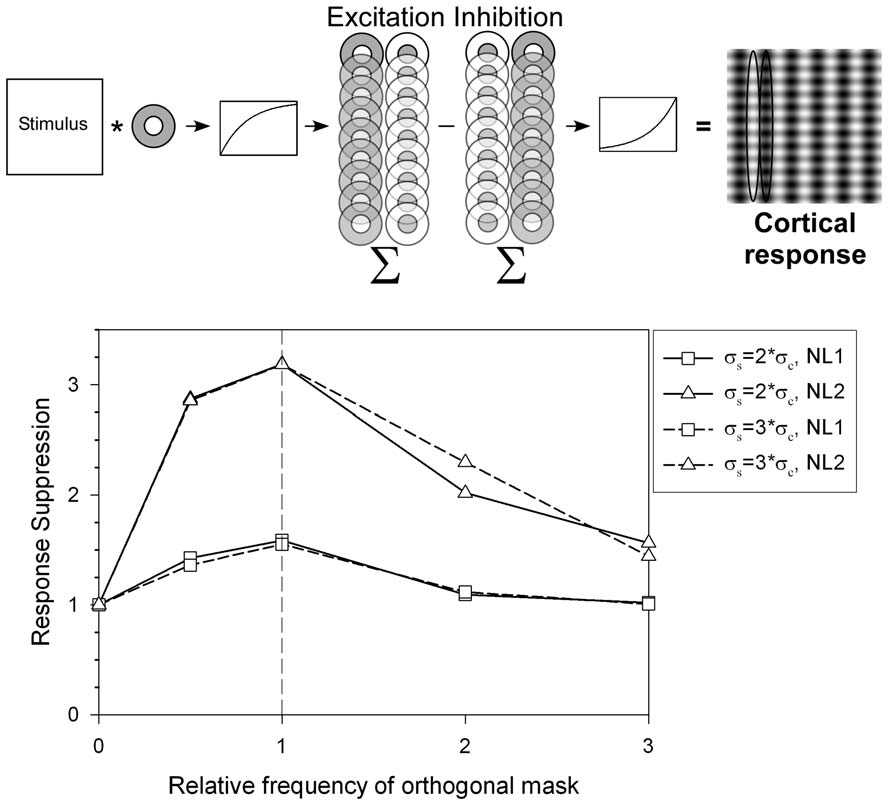
Response suppression in a feed-forward model of cross-orientation suppression as a function of mask frequency. Top: Responses of On- and Off-center LGN cells were convolved with the stimulus, passed through a compressive non-linearity, summed in excitatory and inhibitory push-pull form, and passed through an expansive cortical spike threshold non-linearity. Bottom: Response suppression of the model (response to the test grating alone divided by the response to the test plus the orthogonal mask) as a function of the relative frequency of the orthogonal mask. Response suppression was frequency selective for two different center-surround variance ratios (σ_s_ = 2σ_c_ plotted in solid lines and σ_s_ =  3σ_c_ plotted in dashed lines) and two different compressive nonlinearities (k = e in Equation 2 for NL1 plotted in squares, and k = max(abs(L)) in Equation 2 for NL2 plotted in triangles).

To test the frequency-selectivity of COS in the models of Li et al. ([22]) and Priebe and Ferster ([23]), we computed cortical responses to a vertical test grating in the presence of superimposed horizontal masks of the same or different frequency. The model simulates responses of a simple cell as determined by excitation of LGN cells tuned to the spatial frequency of the test grating (Figure 3). The receptive field of each ON-center cell is modeled as the difference of two Gaussians:
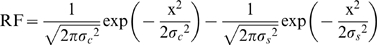
(1)where σ_c_ is the variance of the central Gaussian, and σ_s_ is the variance of the surround Gaussian. OFF-center receptive fields were modeled as negatives of ON-center receptive fields. Linear outputs of LGN cells at each location of the stimulus were approximated by convolving ON- and OFF-center receptive fields with the stimulus (either a single vertical grating, or a vertical grating added to a horizontal mask). The outputs were then subjected to a compressive contrast nonlinearity in the LGN ([22], [23]) of the form:
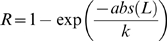
(2)where *R* is the compressed response, L is the linear response, and the value of k dictates the strength of the compression (greater compression for greater values).

Excitation and inhibition in the cortical simple cell receptive field has been modeled by summed responses of LGN cells in “push-pull” form ([22]). Excitation from ON-center LGN cells and inhibition from OFF-center LGN cells form an ON sub-region of the simple cell receptive field, while excitation from OFF-center LGN cells and inhibition from ON-center LGN cells form an OFF sub-region of the simple cell. Summed excitatory and inhibitory responses are then squared, representing an accelerating cortical spike-voltage non-linearity. (A range of different expansive nonlinearities yielded the same qualitative patterns in our simulation.) This model simple cell gives null responses to horizontal (mask) gratings in isolation.

Responses of the model to the test grating plus the mask were computed for masks that were the same frequency as the test, or half, twice, and three times the frequency of the test. We defined response suppression as the response to the grating alone divided by the response to the grating plus the mask.

The graph in the bottom of Figure 3 plots response suppression as a function of the frequency of the orthogonal mask relative to the frequency of the test. The points at zero mask frequency represent model responses to the vertical test alone. To test the generality of the simulations, we implemented two different center-surround variance ratios and two different compressive nonlinearities. Each of the four curves represents one combination of these variables: solid lines represent conditions in which the variance of the surround of the LGN cells is twice the variance of the center, dashed lines represent conditions in which the variance of the surround is three times the variance of the center. Square symbols represent NL1 conditions in which k = e in the compressive nonlinearity (Equation 2), and the triangles represent the more compressive NL2 conditions in which k = max(abs(L)). All combinations of receptive fields and nonlinearities lead to frequency selectivity, with suppression greatest when the frequency of the mask matches that of the test. Increasing the surround variance acts to slightly broaden the frequency tuning, and increasing the strength of the compression acts to increase the overall suppression and sharpen the frequency tuning. The magnitudes of suppression reported in Experiment 1 fell between the suppression values for the two model nonlinearities.

## Discussion

The suggested roles of COS in visual encoding have included orientation tuning ([29], [10], [30]), contrast gain control ([31], [32], [11], [33], [34]), and redundancy reduction in the coding of natural images ([35], [36], [37]). Here we postulate a potential role for COS in the decoding of 3-D slant. We have shown that when textured surfaces are slanted, the release of COS makes the critical orientation flows more visible, which correlates with better perception of 3-D slant. We have shown that COS is frequency specific, and that this specificity can arise in simple feed-forward models of COS. To our knowledge, feed-forward explanations of frequency selectivity of COS have not been suggested previously. The LGN models of COS were formulated on the basis of cat physiology, where almost all cell response functions are compressive as a function of contrast. In primate LGN, M-cells are compressive, but P-cells are fairly linear. Our model thus provides the M-cell component of COS. Since V1 cells get input from P and M-cells, some component of COS involves cortical interactions ([24]).

Purpura et al. ([38]) examined whether neurons in V1 and V2 facilitate the extraction of 2-D orientation patterns for the perception of 3-D shape. Of the 29 neurons in macaque V1 and V2 that were isolated from tetrode recordings, flat plaids induced significant suppression in 78% of the neurons compared to optimal single gratings. Suppression was significantly reduced in 45% of the neurons for plaids slanted along or orthogonal to the optimal orientation. In addition, 28% of V1 and 56% of V2 neurons showed enhanced responses to orientation flows per se, indicating that asymmetries may be more prominent in the 2-D structure of V2 receptive fields. Since COS and surround suppression significantly reduce responses to patterns in natural scenes, stimuli that undermine these sources of suppression may allow V1/V2 to mark areas that have a higher probability of containing 3-D shape. In particular, release from cross-orientation suppression serves to enhance the visibility of orientation flows that are the keys to decoding 3-D shapes as signaled by texture ([3], [2], [5]), shading ([39]), and specular reflection ([40]).
